# Supplementary figures and images for: Adaptive immune defense prevents Bartonella persistence upon trans-placental transmission
Source: PLoS Pathog. 2022 May 17;18(5):e1010489. doi: 10.1371/journal.ppat.1010489 (PMC9113594; doi:10.1371/journal.ppat.1010489)

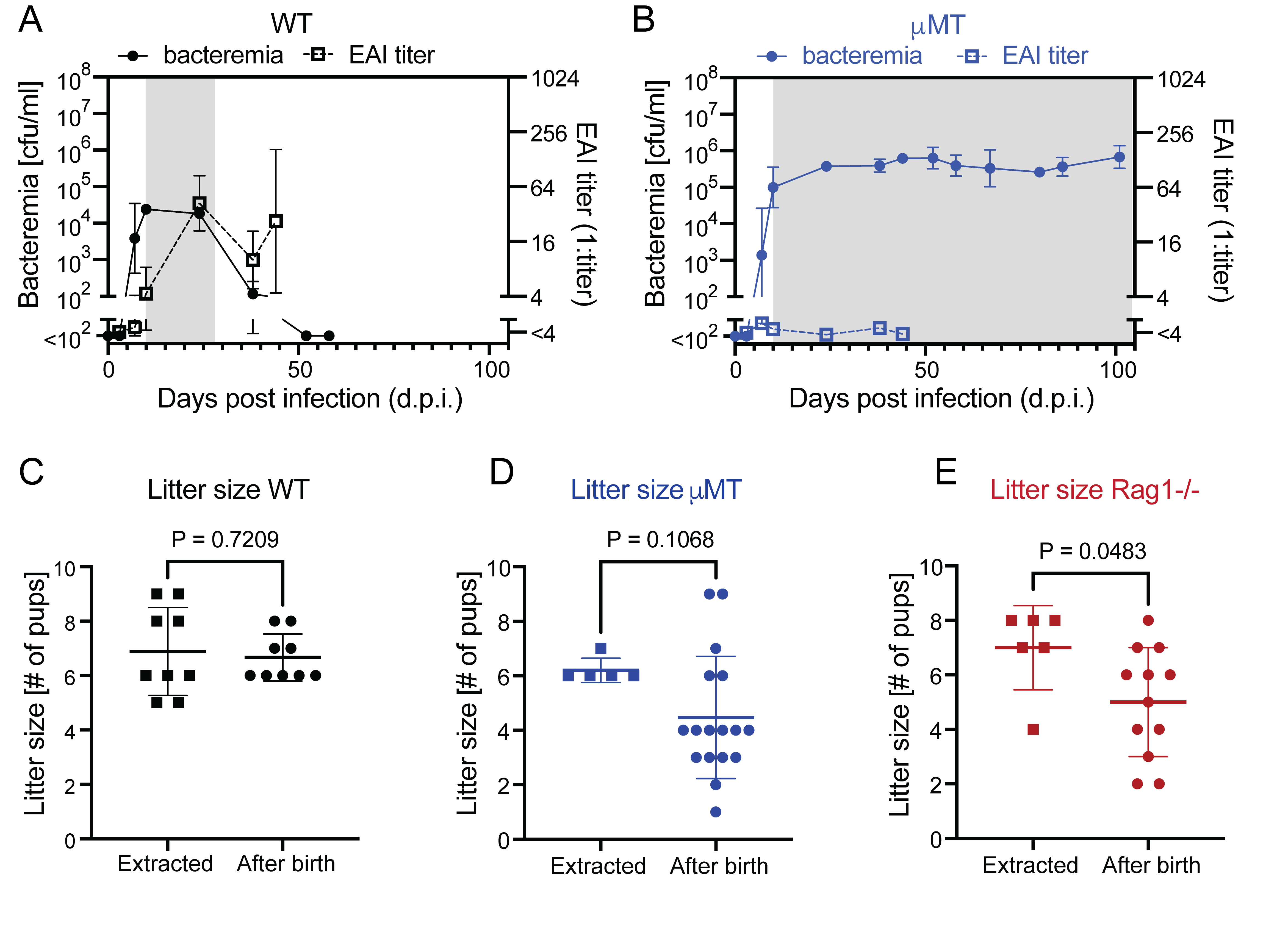

Supplement: S1 Fig — (A-B) We infected female WT (A) and μMT (B) mice and determined bacteremia and erythrocyte adhesion inhibiting (EAI) antibody titers at the indicated time points. The mice and bacteremia curves are the same ones as displayed in Fig 1B. Symbols represent the mean ± SEM of three mice per group. One representative of three experiments is shown. (C-E) We infected WT (C), μMT (D) and Rag1-/- (E) dams with 107 cfu of B. taylorii i.d. and mated them with partners of the same genotype 10 days later (analogously to the experiment in Fig 1). The size of litters was recorded at the time point of embryo extraction on embryonic day 18 (“extracted”) or within 24 h after birth (“after birth”). Symbols in (A-B) show mean ± SD of combined data from two independent experiments with at least three mice per group. (C-E) Each symbol represents an individual litter, horizontal lines and error bars depict the mean ± SD. Statistical analysis was performed by unpaired Student’s t-test, with P-values indicated in the figure. (TIF) [file ppat.1010489.s001.tif]
