# Supplementary material for: Adaptive immune defense prevents Bartonella persistence upon trans-placental transmission
Source: PLoS Pathog. 2022 May 17;18(5):e1010489. doi: 10.1371/journal.ppat.1010489 (PMC9113594; doi:10.1371/journal.ppat.1010489)
Supplement: Attachment — Submitted filename: Siewert_PLoS_Point-by-point_reply.docx [file ppat.1010489.s003.docx]

**Point-by-point reply to reviewers comments**

***Part I - Summary*** *Please use this section to discuss strengths/weaknesses of study, novelty/significance, general execution and scholarship.*

***Reviewer #1:*** *This study explores the role of humoral immune tolerance versus anti-bacterial immunity in vertical/maternal transmission and persistence of Bartonella in mice. The findings of this study reveal a key role for humoral immune defense by the mother and offspring in preventing and eliminating vertical transmission of Bartonella taylorii in mice. These are interesting and novel contributions to the knowledge of the immune response to Bartonella infections.

The questions explored here are well studied and documented. They are of interest to the Bartonella and bacteriology research community, and within the scope of PLOS Pathogens.*

***Reviewer #2:*** *State of the art story excluding vertical transmission of B. taylorii in mice.*

***Reviewer #3:*** *In this work, Siewert et al have investigated the frequency of vertical transmission of Bartonella as a function of the ability to generate Bartonella-neutralizing antibodies of both mothers and their offspring. B-cells and antibodies are shown to be required to limit bacteremia and placental bacterial burden in the mothers on the one hand, and is additionally required to successfully fend off infection in the offspring. The data refute a previous model whereby persistent Bartonella infection was due to immune tolerance to the infection of the offspring. The data are strong and the experiments well controlled.*

We thank the three reviewers for their positive evaluation of our study.

***Part II – Major Issues: Key Experiments Required for Acceptance*** *Please use this section to detail the key new experiments or modifications of existing experiments that should be absolutely required to validate study conclusions.

Generally, there should be no more than 3 such required experiments or major modifications for a "Major Revision" recommendation. If more than 3 experiments are necessary to validate the study conclusions, then you are encouraged to recommend "Reject".*

***Reviewer #1:*** *None*

***Reviewer #2****: - none -*

***Reviewer #3:*** *While concise, the data as currently presented could be improved by the addition of a bit more detail on the following aspects:
1. What is the role of T-cells? The consistent difference in bacterial burden and transmission efficacy between Rag1-/- and uMT mice suggests that T-cells have a role as well. What is the phenotype of T-cell deficient mice in this system?*

In response to the reviewer’s question we have included in our manuscript’s discussion section a paragraph delineating some obvious limitations of our study. Specifically, we acknowledge: “Limitations of our study consist in the largely undefined role of maternal and fetal T cells in vertical *Bartonella* transmission. Higher bacterial loads in Rag1-/- as compared to µMT-/- mothers likely explain a trend towards higher transmission rates in the former and suggest the role of T cells in *Bartonella* control extends beyond the mere help to B cells.”

*2. Which antibody class is most important in humoral defense? Please use ko's specifically lacking IgG or IgA to address this question.*

In response to the reviewer’s question the above-mentioned “limitations of study” paragraph explains further: “The contribution of individual immunoglobulin isotypes to the prevention of vertical transmission and to the control of bacteremia in the offspring, respectively, have not been addressed by our study either. While the IgM response of activation-induced deaminase- (AID-) deficient mice has been shown to effectively control *B. taylorii* in adult mice without a need for isotype class switch (Siewert et al. 2021), the ability of IgG to cross the placenta may improve its antibacterial efficacy at the maternal-fetal interface.”

*3. How important are milk antibodies in preventing bacteremia in infected offspring? Swapping of litters from infected to naive dams, depriving the offspring of potentially protective IgA in milk, would address this question.*

In response to the reviewer’s question the “limitations of study” paragraph specifies: “Last but not least the lack of transplacental *Bartonella* transmission by WT mothers in our model precluded us from analyzing a potential role of breast milk antibodies in preventing bacteremia in the offspring.”

At the outset of our investigation, when failing to detect *Bartonella* infection in WT offspring born to infected WT mothers, we indeed considered the possibility that breast milk antibodies may prevent vertical transmission. We therefore performed an experiment as suggested by the referee, in which WT offspring born to an infected mother were nursed by an uninfected foster mother (litter swap immediately after birth). The result of this experiment was disappointing to us at the time since none of the seven offspring from the swapped litter exhibited any trace of *Bartonella* infection either (data not shown). Fortunately, however, this finding prompted us to investigate bacterial loads in extracted embryos. As reported in Fig. 2C and Tbl. I not one single WT embryo out of 48 embryos analyzed from seven litters was infected, whereas infection rates in µMT and Rag1-/- embryos were substantial. This demonstrated that maternal antibody defense prevented bacterial transmission *in utero*, which seems the predominant if not only route of vertical transmission in our infection model. Peri-natal or post-natal transmission from mother to offspring does not seem to occur in µMT and Rag1-/- animals an any appreciable rate either, since infection rates in 4-week-old µMT and Rag1-/- animals are lower than in extracted embryos of the respective strain (Fig. 1C). We acknowledge, however, the possibility that the apparently exclusively transplacental transmission of *Bartonella* may be inherent to our model and that a potential role of breast milk antibodies in preventing peri-natal or post-natal *Bartonella* transmission to newborn animals remains to be addressed.

Repeating and thereby extending the limited data on litter swap would unfortunately require us to file / renew our respective ethics protocol, which can take an unpredictable but likely significant amount of time, followed by additional experimentation, which will take at least 70 days. In light of this foreseeably significant delay, the negative preliminary data at hand and the above considerations indicating such experiments are unlikely to provide the type of insights hoped for, we regret but have to conclude that such work lies outside the scope of the present revision.

*4. Finally, how effective are antibodies in WT offspring of heterozygous uMT parents at successfully fending off transmission?*

Heterozygous µMT mice have a normally sized B cell compartment (Kitamura *et al.* 1991 Apr 4;350(6317):423-6) and thus are phenotypically wildtype. An experimental setting of WT offspring born to heterozygous µMT parents as cited by the referee is, therefore, expected to phenocopy the setting of WT mothers giving birth to WT offspring studied in our paper and shown not to result in Bartonella transmission.

***Part III – Minor Issues: Editorial and Data Presentation Modifications*** *Please use this section for editorial suggestions as well as relatively minor modifications of existing data that would enhance clarity.*

***Reviewer #1:*** *L 120 – the word "mating" is more appropriate than "impregnation".*

The manuscript’s text has been adapted as recommended by the reviewer.
*L 153 – "bacterial loads" should be used instead "bacterial titers".*

The manuscript’s text has been adapted as recommended by the reviewer.

*L 246 – should be suggests.*
The manuscript’s text has been adapted as recommended by the reviewer.

*L – 256 – I strongly suggest the authors to add the words "although not significant" after the phrase "and a similar trend". In addition, in line 258 the authors should change to "….and possibly also in µMT-/- mice but not in WT….", as P=0.7209 is not considered significant (although stated by the authors as a trend) – otherwise it might be considered as overestimation of the test results.*

Following the reviewer’s recommendation we have inserted “although not statistically significant” in the sentence formerly starting on line 256. It now reads: “We observed that Rag1-/- litters on the day after birth were smaller than at the time point of embryo extraction (E18), and a similar trend, although not statistically significant, was noted for µMT-/- litters but not for WT litters.”

The sentence describing these findings in the Results section (starting line 139) has also been modified accordingly and specifies the *P*-value, on which our statement is based: “We noted that litters of infected Rag1‑/- dams were significantly larger at the time point of embryo extraction than observed on the day after birth and a similar trend, although not statistically significant (p=0.1068), was noted for litters of µMT mothers but not for WT controls (Fig. S1C-E), […].” P=0.7209 describes the comparison of litter sizes between infected and uninfected WT mothers, not µMT mothers as suggested by the referee’s comment, and was stated as not different throughout the manuscript.

We acknowledge that the original sentence on former line 258 was potentially misleading and could be interpreted to state that the perinatal fitness of Bartonella-infected µMT mice was impaired, while we merely intended to refer to µMT mice as a genotype allowing vertical transmission. To avoid potential misinterpretations by the reader the sentence has been modified: “This finding may indicate that congenital *Bartonella* infection may negatively impact the offspring’s perinatal fitness.”, without direct reference to µMT mice.

*L 262-3 – Authors conclude :" Taken together the present findings demonstrate that B cells represent a dual layer of immune protection against vertical transmission of Bartonellae". Authors should be more accurate regarding their findings as their findings are restricted to B. taylorii in mice. I therefore strongly recommend changing the sentence to the following :" Taken together, the present findings demonstrate that B cells represent a dual layer of immune protection against vertical transmission of B. taylorii, and possibly of other Bartonella species, in mice".*

The manuscript’s text has been adapted as recommended by the reviewer.

*L 286 – authors indicate that "blood was collected from the tail vein" – authors should include the time points, frequency of blood collection and how much blood was drawn from each mouse in each time-point. This information is important as it may affect the general health/stress condition of the mice.*

In response to the reviewer’s request the respective paragraph of the Methods section has been amended as follows: “At each time point, for which bacteremia is reported in the figures, 50 µl of blood was collected…[…]. An interval of at least one week was respected between blood draws, except for the first two weeks after adult infection (reported in Figs. 1B and S1A,B), during which two weekly blood samples were collected.”

*L205-6 – the authors wrote :"Not only does the maternal B cell response restrict transmission." This is a fragment that cannot stand by its own. It should be merged with the next sentence in the paragraph.*
Following the reviewer’s recommendation we have merged these two sentences, which read now: «Not only does the maternal B cell response restrict transmission but the immunocompetent offspring’s immune defence also clears the infection, protects against reinfection and mounts a functional antibody response upon bacterial re-challenge.”

*L 290 – delete the word days.*
The word “days» has been deleted as recommended by the referee.

*L 401 – delete the word as.*

The word «as» has been deleted following the referee’s recommendation.

***Reviewer #2****: 1. Introduction is too long - please shorten to ~ 66%.*

In response to the reviewer’s recommendation we have shortened the introduction to 67% of its prior length.

*2. line 268-274: please explain: why was a smR mutant used instead of wildtype bacteria? How was this mutant characterized? Are there any other mutations in this particular strain which might artifically influence the experiments?*

In response to the reviewer’s question we have introduced a short paragraph into the Materials and Methods section that reads as follows: “The *B. taylorii* IBS 296 strain was used for this study since it readily establishes robust bacteremia in mice and its immunobiology in mice has been thoroughly characterized (Siewert et al. 2021, Fromm et al. 2022). Moreover, a GFP-expressing variant is available, thereby enabling the functional EAI antibody readout reported in Figs. 3G and S1A,B. Streptomycin resistance (Sm^R^) encoded by a mutation in *rpsL* facilitates targeted genetic manipulation (Schulein et al., 2002), but is not known or expected to impact the bacterium’s behavior or immunobiology in mice in the absence of antibiotic treatment, neither does this strain feature any other genetic alterations that are known or expected to bias the results reported in this study.”

*3. bacterial load was identified by culture from -80°C frozen samples. Although I know that something like this has been published before, I am critical about this procedure. Did the authors compare these data to data obtaind from fresh samples which underwent direct cultivation?*

Erythrocyte lysis is required to release *Bartonellae* prior to plating, since these bacteria travel in the blood of mice virtually exclusively inside erythrocytes. Accordingly, a freeze-thaw step of blood samples is standard in the *Bartonella* research field when determining bacteriemia. In response to the reviewer’s question we have amended the Materials and Methods section as follows: “Samples were frozen at -80°C for erythrocyte lysis and bacterial release, and after thawing were plated […].

*4. Suppl, Fig. 1: Fig. A and B: please give group sizes. Please wrtite out "EAI" as this abbreviation is difficult to find in the text.*

Following the reviewer’s request the revised legends to Fig. 1B and to S1A,B (reporting on the same experiment) specify: “Symbols represent the mean ± SD of three mice per group. One representative of three experiments is shown.” Moreover, “EAI” is written out in the revised legend to Fig. S1: “We […] determined bacteremia and erythrocyte adhesion inhibiting (EAI) antibody titers at the indicated time points.”

***Reviewer #3****: none*
